# A Novel CD147 Inhibitor, SP-8356, Attenuates Pathological Fibrosis in Alkali-Burned Rat Cornea

**DOI:** 10.3390/ijms21082990

**Published:** 2020-04-23

**Authors:** Chanmin Joung, Hyojin Noh, Jeein Jung, Hwa Young Song, Hwanse Bae, Kisoo Pahk, Won-Ki Kim

**Affiliations:** 1 Institute for Inflammation Control, Korea University, Seoul 02841, Korea; joungchanmin@korea.ac.kr (C.J.); nohhj87@korea.ac.kr (H.N.); jeegel@korea.ac.kr (J.J.); 2014250013@korea.ac.kr (H.B.); 2 Shinpoong Pharmaceutical Company, Ansan 15610, Korea; shy997@korea.ac.kr; 3 Department of Nuclear Medicine, Korea University Anam Hospital, Seoul 02841, Korea

**Keywords:** corneal alkali burn, corneal haze, myofibroblast, alpha-smooth muscle actin, collagen type III, matrix-metalloproteinase, MMP-9

## Abstract

The corneal fibrotic responses to corneal damage often lead to severe corneal opacification thereby resulting in severe visual impairment or even blindness. The persistence of corneal opacity depends heavily on the activity of corneal myofibroblast. Myofibroblasts are opaque and synthesize a disorganized extracellular matrix (ECM) and thus promoting opacification. Cluster of differentiation 147 (CD147), a member of the immunoglobulin superfamily, is known to play important roles in the differentiation process from fibroblast to myofibroblast in damaged cornea and may therefore be an effective target for treatment of corneal opacity. Here, we examined the therapeutic efficacy of novel CD147 inhibiting verbenone derivative SP-8356 ((1S,5R)-4-(3,4-dihydroxy-5-methoxystyryl)-6,6-dimethylbicyclo[3.1.1]hept-3-en-2-one) on corneal fibrosis. Topical SP-8356 significantly reduced corneal haze and fibrosis in the alkali-burned cornea. In detail, SP-8356 inhibited both alpha-smooth muscle actin (α-SMA) expressing myofibroblast and its ECM-related products, such as matrix-metalloproteinase-9 and collagen type III and IV. Similar to SP-8356, topical corticosteroid (prednisolone acetate, PA) also reduced the ECM-related products and opacification. However, prednisolone acetate failed to decrease the population of α-SMA-positive corneal myofibroblast. In conclusion, SP-8356 is capable enough to prevent corneal haze by preventing pathological fibrosis after severe corneal damage. Therefore, SP-8356 could be a potentially promising therapeutic drug for corneal fibrosis.

## 1. Introduction

Corneal opacity is a common clinical finding that interferes with the cornea’s light transmission thereby impairing visual function, and it is also a leading cause of blindness globally [1,2]. Corneal fibrosis response to corneal damage from various origins including injury, surgery, infection, chemical burns, or other insults often leads to severe pathologic corneal opacity thereby greatly affecting the patient’s quality of life [2]. Thus, there is a need to develop a therapy against corneal fibrosis, which can prevent the process of corneal opacity or reverse the pre-existing fibrotic tissue to the transparent cornea.

Corneal fibrosis following corneal injury has been closely associated with persistent corneal myofibroblast activation in damaged cornea [3,4,5,6]. Following corneal injury, corneal fibroblast becomes activated and differentiates to myofibroblast [5]. Compare to fibroblast, myofibroblast can generate a disorganized fibrotic extracellular matrix (ECM), which contributes to decreasing in refractory index with increasing light scattering significantly [7,8,9].

Cluster of differentiation 147 (CD147), also known as extracellular matrix-metalloproteinase (MMP) inducer (EMMPRIN), is a well-known transmembrane glycoprotein that induces MMP activation [10]. Accumulating evidence suggests that CD147 promotes tumor growth factor-β (TGF-β) mediated myofibroblast differentiation [11]. Furthermore, CD147 also induces MMP-9 which disrupts epithelial basement membrane thereby boosting both epithelial-stromal cell interaction and penetration of myofibroblast-inducing factors that eventually lead to accelerating myofibroblast differentiation [12,13]. Therefore, CD147 and its subsequent MMP-9 activation could be a potential therapeutic target for attenuation of corneal fibrosis.

Recently, we reported that a novel synthetic small-molecule drug SP-8356 ((1S,5R)-4-(3,4-dihydroxy-5-methoxystyryl)-6,6-dimethylbicyclo[3.1.1] hept-3-en-2-one) directly binds to CD147 thereby inhibiting neointimal hyperplasia and stabilizing plaque vulnerability in animal models through the inhibition of MMP-9 activity [14,15]. Furthermore, we also found that SP-8356 possesses anti-tumor effect through the inhibition of CD147/MMP-9 pathway [16]. In the present study, we studied the inhibitory pharmacological effect of SP-8356 on corneal fibrosis known to be mediated by CD147/MMP-9.

## 2. Results

### 2.1. SP-8356 Improves Corneal Haze after Alkali Burn

**Figure 1:**
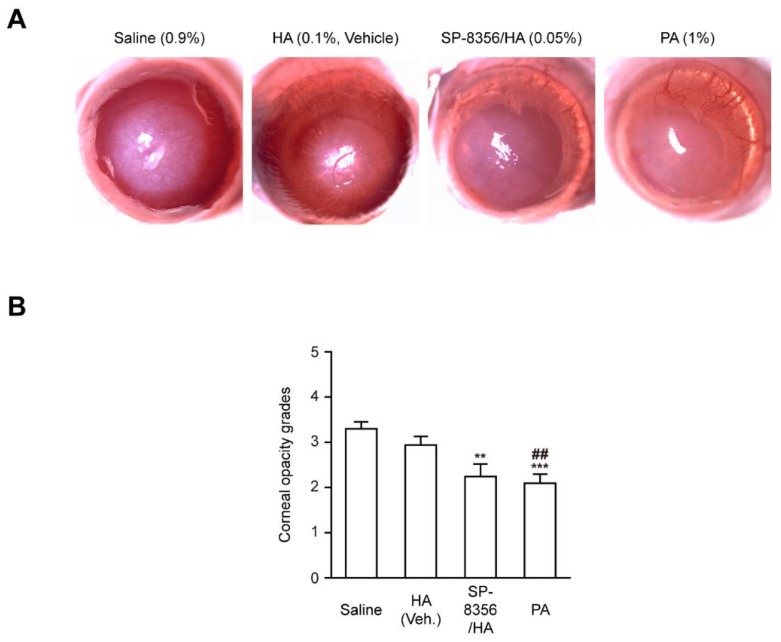
SP-8356 inhibits alkali-induced corneal haze at 2-week after alkali burn. (**A**) Representative images of corneal haze (HA; 0.1% hyaluronic acid, SP-8356/HA; 0.933 mM SP-8356 dissolved in 0.1% hyaluronic acid, PA; 1% prednisolone acetate). (**B**) Quantitative analysis of corneal opacity (*n* = 30 for saline, *n* = 34 for HA, *n* = 33 for SP-8356/HA, *n* = 32 for PA). All values are shown as means ± standard deviation (SD, ** *p* < 0.01 vs. saline. *** *p* < 0.001 vs. saline. ^##^ *p* < 0.01 vs. HA).

Both SP-8356 dissolved in hyaluronic acid (SP-8356/HA) and prednisolone acetate (PA) significantly attenuated the severity of corneal opacity compared to the saline-treated control group in Corneal Alkali Injury (CAI) rat models (Figure 1A,B). Furthermore, the area of opaque region in cornea was also decreased by SP-8356/HA and PA, whereas HA alone could not improve the corneal haze (Supplementary Figure S1).

### 2.2. SP-8356 Depletes Myofibroblast Population in the Alkali-Burned Cornea

**Figure 2:**
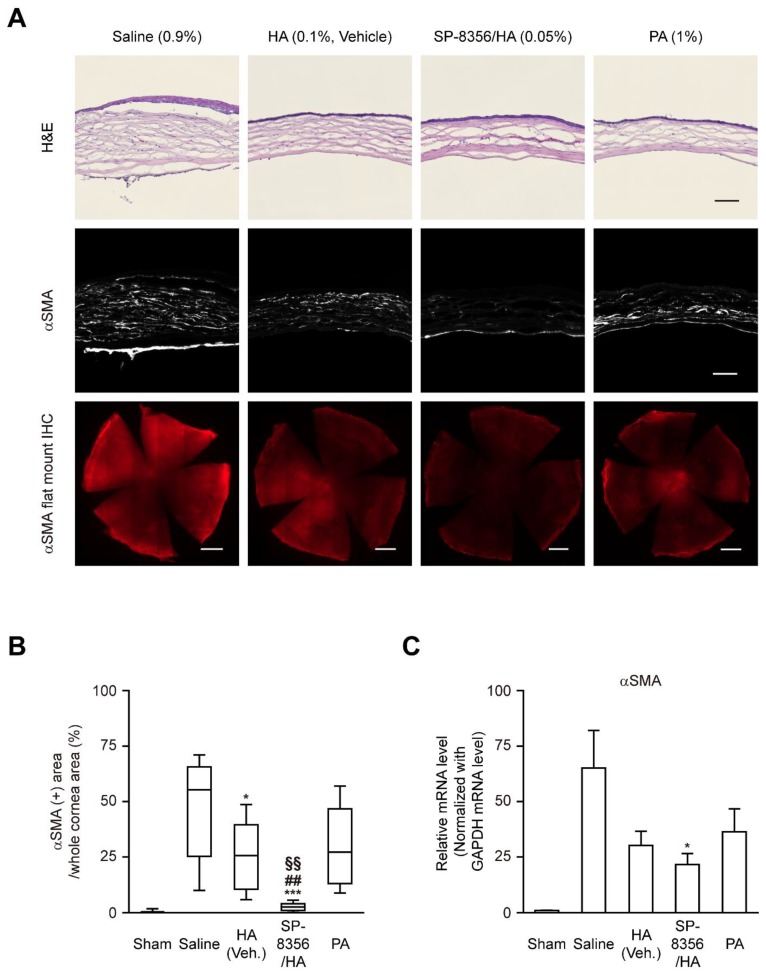
SP-8356 inhibits myofibroblast population in cornea at 2-week after alkali burn. (**A**) Representative images of myofibroblast population. Alkali-burned whole cornea sections were flat-mounted and stained with hematoxylin and eosin (H&E) and anti-αSMA antibody. Scale bars for corneal H&E and immunostaining, 100 μm (magnification, 200×). Scale bar for flat-mounted whole cornea immunostaining, 1 mm. (**B**) Quantitative analysis of αSMA in the whole cornea (*n* = 7 for sham, *n* = 8 for saline, *n* = 10 for HA, *n* = 9 for SP-8356/HA, *n* = 10 for PA). All values are shown as means ± SD (* *p* < 0.05 vs. saline. *** *p* < 0.001 vs. saline. ^##^ *p* < 0.01 vs. HA. ^§§^ *p* < 0.01 vs. PA). (**C**) Quantitative analysis of the relative mRNA level of αSMA (*n* = 9 for sham, *n* = 10 for saline, *n* = 10 for HA, *n* = 10 for SP-8356/HA, *n* = 10 for PA). The mRNA levels are shown as means ± SD (* *p* < 0.05 vs. saline).

It is well known that a sustained population of myofibroblasts increases the expression of alpha-smooth muscle actin (α-SMA) and promotes corneal haze [5,7,17]. The transverse corneal section immunohistochemistry (IHC) showed that SP-8356/HA decreased α-SMA expression in the corneal stroma (Figure 2A). Furthermore, flat-mount IHC images revealed that SP-8356/HA drastically down-regulated the area of the α-SMA (+) region among the whole cornea (Figure 2A,B). The mRNA level of α-SMA in the entire corneal lysate was also significantly reduced in SP-8356/HA-treated cornea (Figure 2C). Although treatment with HA alone reduced α-SMA expression in the alkali-injured cornea, co-treatment with SP-8356 further decreased both α-SMA protein and mRNA level of α-SMA (Figure 2). In addition, treatment with SP-8356 alone depleted the mRNA level of α-SMA in the alkali-injured cornea (Supplementary Figure S2A). However, PA did not show notable effect on depleting either the α-SMA expression in the corneal stroma or the mRNA level of α-SMA in the entire corneal lysate (Figure 2 and Supplementary Figure S2A).

### 2.3. SP-8356 Down-Regulates MMP-9 Activity in the Damaged Cornea

**Figure 3:**
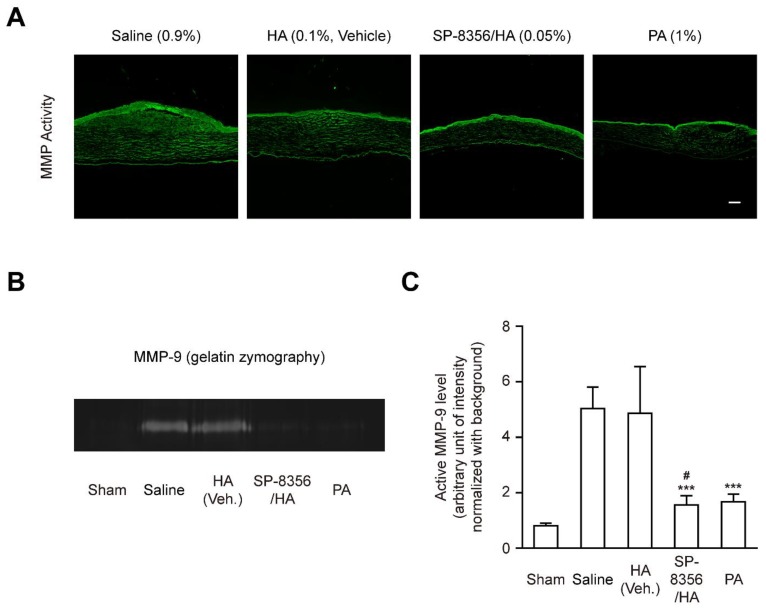
SP-8356 inhibits matrix-metalloproteinase (MMP) activity at 2-week after alkali burn. (**A**) Representative image of MMP activity, which is visualized with in situ zymography. Scale bar, 100 μm (magnification, 200×). (**B**) Representative image of MMP-9 gelatin acrylamide gel zymography. (**C**) Quantitative analysis of the relative level of MMP-9 activity in whole corneal lysates (*n* = 9 for sham, *n* = 12 for saline, *n* = 9 for HA, *n* = 9 for SP-8356/HA, *n* = 10 for PA). MMP-9 activities are shown as means ± SD (*** *p* < 0.001 vs. saline. ^#^ *p* < 0.05 vs. HA).

In situ zymography and gelatin acrylamide gel zymography showed that SP-8356/HA and PA significantly reduced the MMP activities in the cornea (Figure 3). The topical administration of SP-8356 alone also markedly reduced the MMP-9 activity (Supplementary Figure S2B,C).

### 2.4. SP-8356 Suppresses the Synthesis of Pathologic Collagen Subtype

**Figure 4:**
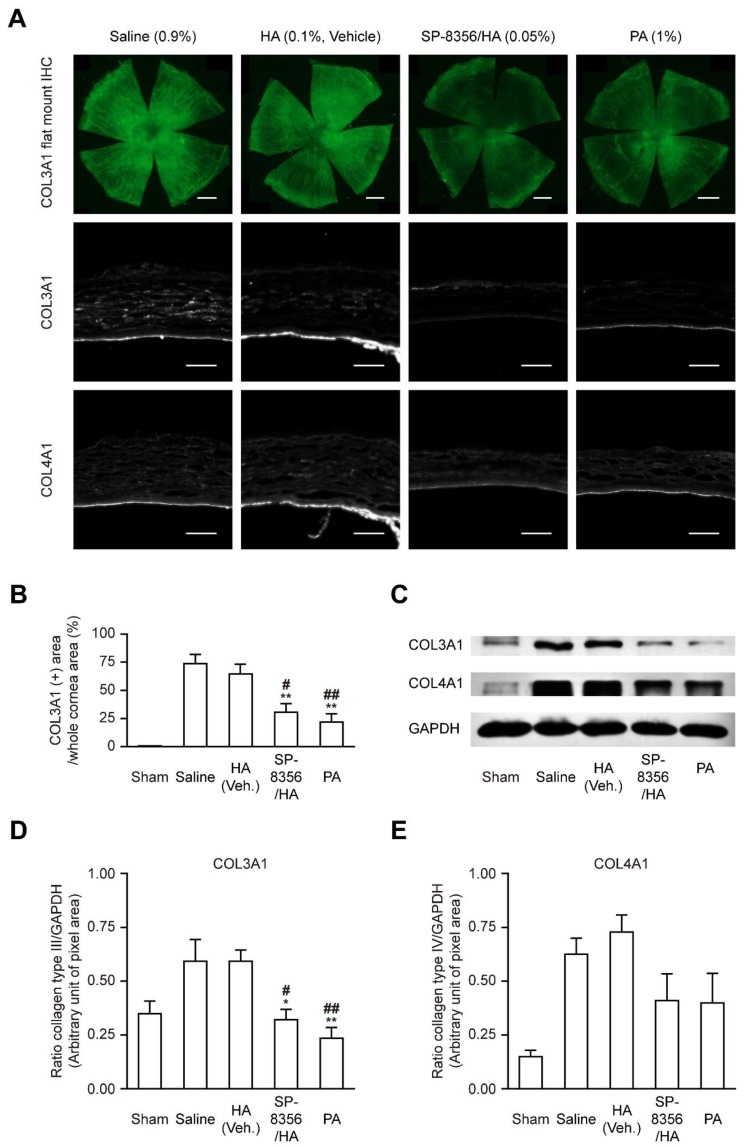
SP-8356 reduces fibrosis-related collagen expression at 2-week after alkali burn. (**A**) Representative images of collagen type III (COL3A1) and type IV (COL4A1) expression. Scale bars for corneal transverse sections IHC, 100 μm (magnification, 200×). Scale bars for flat-mounted cornea IHC, 1 mm. ( ) Quantitative analysis of COL3A1 expression in the whole cornea (*n* = 4 for sham, *n* = 5 for saline, *n* = 8 for HA, *n* = 8 for SP-8356/HA, *n* = 7 for PA). Values are shown as means ± SD (** *p* < 0.01 vs. saline. ^#^ *p* < 0.05 vs. HA. ^##^ *p* < 0.01 vs. HA). (**C**) Effect of SP-8356/HA on COL3A1 and COL4A1 expressions in the whole corneal lysate. (**D**) Quantitative analysis of COL3A1 expression in whole corneal lysates (*n* = 13 for sham, *n* = 11 for saline, *n* = 10 for HA, *n* = 10 for SP-8356/HA, *n* = 9 for PA). Values are shown as means ± SD (* *p* < 0.05 vs. saline. ** *p* < 0.01 vs. saline. ^#^ *p* < 0.05 vs. HA. ^##^ *p* < 0.01 vs. HA). (**E**) Quantitative analysis of COL4A1 expression in whole corneal lysates (*n* = 13 for sham, *n* = 14 for saline, *n* = 10 for HA, *n* = 9 for SP-8356/HA, *n* = 9 for PA). Values are shown as means ± SD.

Of collagen types, type I is a major component of the normal corneal stroma [8]. In damaged cornea, myofibroblast synthesizes massive amount of heterogenous collagens and increment of other collagen subtypes can result in the opaqueness of damaged cornea [8,18,19,20]. Levels of collagen type III and IV (COL3A1 and COL4A1) are typically escalated in damaged cornea and related to corneal haze formation [8,18,21,22,23]. Both SP-8356/HA and PA reduced the COL3A1 expression, whereas COL4A1 expression was not significantly altered by SP-8356/HA or PA treatment (Figure 4). In addition, HA treatment alone failed to reduce both COL3A1 and COL4A1 expressions.

### 2.5. Topical Administration of SP-8356 Reduces Myofibroblast-Inducing Cytokine, TGF-β1

**Figure 5:**
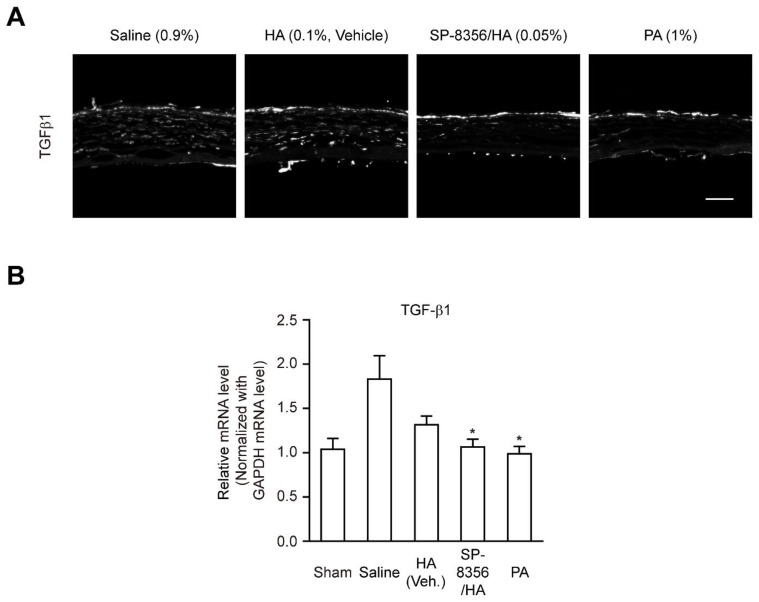
SP-8356 inhibits the expression of TGF-β1 at 2-week after alkali burn. (**A**) Representative images of TGF-β1 expression in the stroma of the alkali-burned cornea. Scale bar, 100 μm (magnification, 200×). (**B**) Quantitative analysis of the relative mRNA level of TGF-β1 (*n* = 6 for sham, *n* = 6 for saline, *n* = 6 for HA, *n* = 6 for SP-8356/HA, *n* = 6 for PA). All mRNA values are shown as means ± SD (* *p* < 0.05 vs. saline).

TGF-β1 has been well known to promote myofibroblast differentiation of fibroblast in the damaged cornea [24,25,26]. Both SP-8356/HA and PA significantly reduced TGF-β1 expression in the damaged cornea, whereas HA treatment alone did not inhibit TGF-β1 expression significantly (Figure 5).

## 3. Discussion

CD147 plays a crucial role in fibrosis processes in response to corneal injury [11,27,28,29,30]. In the present study, we clearly found that a novel CD147 inhibitor SP-8356 markedly reduces corneal fibrosis and haze via inhibition of myofibroblast population and MMP-9 activity in the alkali-burned cornea.

Fibrosis in injured cornea relies on direct cell-cell interaction between the corneal cells [4]. Several previous studies reported that CD147 is involved in this direct cell-cell interaction through contact-dependent (i.e., juxtacrine) signaling between the corneal cells [4,5]. Gabison et al. [10] found that CD147 expression and ensuing MMP activation were up-regulated in corneal fibroblast after contact with epithelial cells. Moreover, exposure of corneal fibroblast to exogenous CD147 extracellular domain induces myofibroblast differentiation and MMP production [11]. In addition to exogenous CD147 which serves as a ligand for fibroblast, endogenous CD147 located in the membrane of corneal fibroblast also contributes to fibrosis. Huet et al. [11] reports that reduced expression of endogenous CD147 attenuates TGF-β dependent myofibroblast differentiation and MMP production. Thus, both exo- and endogenous CD147 are crucial for corneal fibrosis, although the detailed underlying mechanism of CD147 in juxtacrine signaling remains unclear. One possible mechanism is that CD147 can serve as both receptor and ligand for adjacent CD147, which is called the homophilic interaction such as dimerization [13,31,32]. Recently, we found that SP-8356 shows a high binding affinity with CD147 and directly inhibits CD147 dimerization [14]. Thus, the inhibitory effect of SP-8356 on CD147 dimerization may contribute to its anti-fibrotic effect in the alkali-burned cornea.

Previously, Singh et al. [33] reported that inhibition of TGF-β1 signaling pathway reduced α-SMA expressing myofibroblasts in a paracrine signal-dependent manner, whereas α-SMA positive cells were slightly reduced in juxtacrine and paracrine signal-dependent manners between stromal fibroblasts and bone marrow-derived cells. Considering the higher frequency of contact-dependent (juxtacrine) signaling between corneal fibroblast and other cell types in injured cornea [10,33,34]. Thus, the blockade of TGF-β1 alone may not be sufficient to reduce myofibroblast population in injured cornea, and the concurrent blockade of juxtacrine signaling pathway may be crucial for attenuation of myofibroblast activity. As CD147 is a key mediator of the juxtacrine signaling pathway in injured cornea, the profound therapeutic effect of SP-8356 on corneal fibrosis could be explained by its high binding affinity to CD147 and suppression of CD147 induced signaling pathway.

Presently, topical corticosteroids are frequently used to prevent corneal haze in clinical fields and PA is one popular topical steroid [5,35,36,37,38]. Thus, we used PA as a positive control drug. In the present study, both SP-8356 and PA decreased TGF-β1 expression (Figure 5). However, only SP-8356 depleted α-SMA (+) myofibroblast population in injured cornea whereas PA slightly decreased the number of myofibroblasts and α-SMA expression (Figure 2). Similarly, Hindman et al. and Kim et al. [38,39] reported that PA decreased the number of myofibroblast in damaged cornea, but this reducing effect disappeared when PA treatment is discontinued. In addition, Hill et al. [40] reported that corticosteroid is ineffective to eliminate myofibroblast population in damaged cornea. Thus, PA seems to improve corneal haze regardless of depletion of myofibroblast population (Figure 1 and Supplementary Figure S1). Furthermore, several previous studies report that discontinuation of PA led to recurrence or exacerbation of corneal haze [41,42]. In addition, chronic use of PA is limited in clinical field due to its unwanted adverse effect such as elevation of intraocular pressure which eventually results in keratoconus or glaucoma [43,44,45]. Thus, development of a novel non-steroidal anti-fibrotic drug on corneal haze is required and SP-8356 could be a strong candidate for this purpose.

HA itself has an ameliorative effect that can heal corneal epithelial defect, thereby widely being used for dry-eye syndrome and corneal epithelial disorders [46,47,48]. In addition, the viscoelastic properties of HA extend its residence on the corneal surface [49]. Thus, in the present study we treated SP-8356 in combination with diluted HA to prolong the ocular residence time of SP-8356. Treatment with HA alone significantly decreased the expression of α-SMA in alkali-burned cornea. In addition, this alleviating effect was further enhanced by the combined treatment with SP-8356. Treatment with HA alone could not suppress corneal haze, pathologic collagen synthesis, and MMP activation but reduced them by co-treatment with SP-8356. Moreover, the anti-fibrotic effect of SP-8356 was observed in the absence of HA. Thus, the topical administration of SP-8356 in combination with HA can effectively attenuate the corneal haze and its related corneal fibrosis.

Taken together, topical administration of SP-8356 inhibits corneal haze and associated myofibroblast differentiation, possibly via inhibiting CD147 binding and ensuing MMP-9 activation. Furthermore, SP-8356 exhibits a superior anti-fibrotic effect on damaged cornea compared to corticosteroid (PA). Although further study is warranted to elucidate the detailed underlying mechanism of anti-fibrotic effect of SP-8356 in damaged cornea, SP-8356 could be a potential therapeutic agent for pathologic corneal fibrosis which eventually leads to corneal haze.

## 4. Materials and Methods

### 4.1. Animals

Six weeks old Male Sprague-Dawley (SD) rats were obtained from Orient Bio (Seongnam, Korea). All rats were housed under a 12-h light/dark cycle with free access to water and food. All experimental protocols were approved by the Institutional Animal Care and Use Committee of Korea University College of Medicine (Approval No. KOREA-2018-0030).

### 4.2. Corneal Alkali Injury (CAI) Model

After the 2-weeks of acclimation, 8-week old rats were subjected to corneal alkali burn as described previously [50]. In brief, rats were anesthetized with 3.5% isoflurane in a 2:1 N_2_O/O_2_ mixture. The gas mixture was maintained in the anesthesia chamber via rat’s inhalation through a 2.5% nasal cone. A circular 3 mm diameter filter paper soaked in 1 N NaOH was adhered to the center of the cornea for 1 min. Subsequently, corneas were rinsed with 30 mL of 0.9% saline. All CAIs were induced in the right eyes. After induction of alkali burn, all rats received 50 μL of topical administration of antibiotics (5 mg/mL levofloxacin, Santen; Osaka, Japan) twice a day until when the rats were euthanized, to prevent unintentional infection. The rats were randomly subdivided in to four groups. The saline group was topically treated with 50 μL of 0.9% saline twice a day and the HA group was topically treated with 50 μL of 0.1% sodium hyaluronate (Xenobella 0.1 SD, Chong Kun Dang; Seoul, Korea) twice a day. The SP-8356/HA group was topically treated with 50 μL of 0.05% (w/v, 933 μM) SP-8356 dissolved in 0.1 % sodium hyaluronate twice a day and the PA group was topically treated with 50 μL of 1% prednisolone acetate (Pred Forte, Allergan; Dublin, Ireland) twice a day. To exclude the efficacy of HA, 0.2X phosphate-buffered saline (PBS) buffer, which was prepared from 1X PBS (137 mM NaCl, 2.7 mM KCl, 4.3 mM Na_2_HPO_4_, 1.4 mM KH_2_PO_4_, pH 7.2, Biosesang; Seongnam, Korea) diluted 1:4 ratio with 0.9% saline, was used as a vehicle (Supplementary Figure S2). The 0.2X PBS group was topically treated with 50 μL of 0.2X PBS twice a day. The SP-8356 group was topically treated with 50 μL of 0.05% (w/v, 933 μM) SP-8356 dissolved in 0.2X PBS twice a day. For evaluation of cytokine expression in cornea after damage, rats were euthanized five days after CAI. For evaluation of corneal haze and fibrosis induced by alkali burn, rats were euthanized 2-week after CAI.

### 4.3. Macroscopic Images of CAI Eyes

To capture the macroscopic images of eyes, rats were anesthetized with an intraperitoneal injection of 1 mL ketamine (Yuhan Ketamine 50 Inj., Yuhan; Seoul, Korea) and xylazine hydrochloride (Rompun^®^ Inj., Bayer; Leverkusen, Germany) mixture in 10:3 ratio. The images were taken with charge-coupled device (CCD) camera (AcquCAM 23GR, JNOpTIC Co; Seoul, Korea). The sets of eye images captured at various focused regions were staked and vertically merged into single image using Photoshop CC 2018 software (Adobe; San Jose, CA, USA).

### 4.4. Assessment of Corneal Opacity

The opacity grade of the cornea was determined using the scoring system by Sonoda and Streilein [51] and the opacity score between 0 to +4 was graded as previously described [52]. Two independent researchers (CJ and KP) scored the opacity grade in a blinded manner.

### 4.5. Tissue Preparation

Rats were sacrificed humanely using the CO_2_ chamber, and eyeball of rats was dissected for separation of corneal tissue. Corneas were fixed in 4% paraformaldehyde (PFA, Biosesang) for 12 h at 4 °C with shaking, washed in 1X PBS overnight at 4 °C with shaking, cryoprotected in 30% sucrose in 0.1 M PB buffer for 12 h at 4 °C with shaking. Corneas were embedded in optimal cutting temperature (OCT) compound (Scigen Scientific; Gardena, CA, USA), and stored at −80 °C until cryosection was performed. Transverse serial sections of 8 μm thickness were prepared with a cryostat (CM3050S, Leica; Wetzlar, Germany) and collected on silane-coated glass slides (5116-20F, Muto Pure Chemicals; Tokyo, Japan). Samples were stored at −20 °C until IHC and hematoxylin and eosin (H&E) staining were performed.

### 4.6. H&E Staining

H&E staining of frozen cornea cryosections was performed by following procedure: (i) corneal samples were dried for 1 h at room temperature and washed with 1X PBS, (ii) washed with distilled water, (iii) immersed and stained in hematoxylin solution (Harris modified) for 2 min, (iv) washed with distilled water 10 times, (v) immersed and counterstained in eosin solution for 2 min, (vi) washed with distilled water 10 times, (vii) washed and dehydrated in 50% ethanol for 10 sec, (viii) washed and dehydrated in 70% ethanol for 10 sec, (ix) washed and dehydrated in 90% ethanol for 10 s, (x) washed and dehydrated in 100% ethanol for 10 s, twice, (xi) dipped in xylene for 10 s, (xii) mounted with Canada balsam (Junsei; Tokyo, Japan) dissolved in xylene. All H&E staining images were taken with Zeiss Axio Scan.Z1 (Carl Zeiss; Jena, Germany).

### 4.7. IHC

IHC of frozen cornea cryosections was performed by following procedure: (i) corneal samples were dried for 1 h at room temperature and washed with 1X PBS twice, (ii) blocked with blocking buffer composed of 5% normal goat serum (NGS) (Vector Laboratories; Burlingame, CA, USA) and 0.1% Triton X-100 (Sigma-Aldrich) in 1X PBS for 1 h at room temperature, (iii) incubated in the primary antibody solution at 4 °C overnight, (iv) washed with 1X PBS twice, (v) incubated with secondary antibodies conjugated with Alexa 555 or 488 (Invitrogen; Carlsbad, CA, USA) fluorescent dye in 5% NGS-based blocking buffer for 1 h at room temperature. (vi) washed with 1X PBS twice, (vii) mounted with mounting medium (DAKO; Santa Clara, CA, USA). The primary antibodies used for IHC were anti-α-SMA (1:400 dilution, ab7817, Abcam), anti-collagen type III (1:400 dilution, ab7778, Abcam), anti-collagen type IV (1:400 dilution, ab6586, Abcam), and anti-TGF-β1 (1:500 dilution, ab92486, Abcam) antibodies. All fluorescence images were taken with Zeiss Axio Scan.Z1 (Carl Zeiss; Jena, Germany).

### 4.8. Flat-Mounted Cornea IHC

Flat-mounted cornea IHC of corneas was performed by following procedure: (i) fixed corneas were washed with 1X PBS for 24 h at 4 °C with shaking, (ii) transferred to 1% Triton X-100 (Sigma-Aldrich) in 1X PBS (1% PBST) and incubated for 24 h at 4 °C with shaking, (iii) immersed in 5% NGS-based blocking buffer for 24 h at 4 °C with shaking, (iv) immersed in fluorophore-conjugated primary antibody solution at 4 °C for 48 h with shaking, (v) washed with 1% PBST for 1 h at room temperature with shaking twice, (vi) Specimens were placed on a slide glass and mounted with mounting medium (ProLong^TM^ Gold, Invitrogen). Anti- α-SMA-Alexa 647 (1:100 dilution, sc-53015-AF647, Santa Cruz) and anti-COL3A1-Alexa 488 (collagen type III, 1:100 dilution, sc-271249-AF488, Santa Cruz) antibodies were used for flat-mount IHC. All fluorescence images were taken with Zeiss Axio Scan.Z1 (Carl Zeiss). The area of collagen type III and α-SMA expressed region was quantified with threshold-based area measuring method using Zeiss Efficient Navigation (ZEN) software (desk, version 2.3, Carl Zeiss).

### 4.9. Evaluation of the Area of Opaque Region in Cornea

To evaluate the area of opaque region in whole cornea, bright field images of flat-mount corneas were captured. In detail, overhead projector (OHP) film printed black with laser printer was placed below the slide glass. 3 mm depth of region was imaged under 40× magnification with 40 μm intervals. The images were captured with Edge 3D microscope (Edge-3D; Paia, HI, USA) and mounted on D5500 digital camera (Nikon; Tokyo, Japan). The sets of corneal images were staked and vertically merged into the single image using Edge panfocal software 2.7.4 (Edge-3D). The area of opaque region was graded by following procedure: (i) the flat-mounted cornea was divided into five regions of interest (ROI, quadrants, and center, Supplementary Figure S1) (ii) the area of opaque region was evaluated based on the visual scoring system (0; Clear, detail of printed black pattern is visible through cornea and boundary of vacant pore are not scattered by cornea; 1+, less than 30% of region is opaque; 2+, more than 30% of region is opaque) (iii) scores of five ROIs were summed and used as representative score of each CAI rat. Two independent researchers (CJ and KP) evaluated the grade in a blinded manner.

### 4.10. In Situ MMP Zymography

Unfixed corneas were prepared for cryosection. In brief, unfixed corneas embedded in OCT compound (Scigen Scientific) and froze with isopentane filled container immersed in liquid nitrogen. Transverse serial sections of 8 μm thickness were prepared with a cryostat (CM3050S, Leica) and collected on silane-coated glass slides (5116-20F, Muto Pure Chemicals) within 1-week after harvest. In situ MMP zymography was performed by following procedure: (i) corneal sections were dried at room temperature for 1 h, (ii) the substrate containing DQ gelatin fluorescein conjugate (Invitrogen), which diluted a 1:50 ratio with zymogram developing buffer (Invitrogen), was prepared, (iii) corneal sections were rehydrated, (iv) incubated in the substrate for 12-h at 37 °C humidity chamber, (v) washed with 1X PBS three times, (vi) mounted with mounting medium (DAKO). All gelatinase activity images were taken with Zeiss Axio Scan.Z1 (Carl Zeiss).

### 4.11. Western Blot Analysis

Corneas of sacrificed rats were harvested in ice-chilled 1× PBS and lysed in RIPA buffer (Thermo Fisher Scientific; Waltham, MA, USA) with protease inhibitor (Gendepot; Katy, TX, USA). Using the BCA protein assay kit (Thermo Fisher Scientific), protein concentrations of corneal lysates were quantified. Then the lysates with 20 μg of protein mixed with SDS sample buffer and heated for 5 min at 99 °C. Proteins were separated via SDS-PAGE at 200 V for 90 min and transferred to nitrocellulose membrane and blotted with anti-COL3A1 (1:100 dilution, sc-271249, Santa Cruz), anti-collagen type IV (1:2000 dilution, ab6586, Abcam), and anti-GAPDH (1:2000 dilution, MA5-15738, Thermo Fisher Scientific) antibodies. Subsequently, goat anti-mouse HRP and goat anti-rabbit HRP antibodies (1:20,000 dilution, 31430, 31460, Thermo Fisher Scientific) were used. Protein bands were detected by using ECL reagents (GE Healthcare; Marlborough, MA, USA). The images of films were captured with Bio 5000 scanner (Microtek; Hsinchu, Taiwan). The quantification of protein band size was analyzed with ImageJ open source software (version 1.45s, National Institute of Health, NIH; Bethesda, MD, USA).

### 4.12. Gelatin Zymography

The activity of MMP-9 in damaged corneas was evaluated by gelatin zymography. The lysates of unfixed corneas were prepared with identical ways described in the western blot method. The lysates with 10 μg of protein were intermingled with a non-reducing loading buffer and subsequently transferred to 10% gelatin acrylamide gels without the heating process. The gels were comprised of 1 mg/mL gelatin powder (JT Baker Chemical Co.; Phillipsburg, NJ, USA) and 0.4% glycerol (Sigma-Aldrich) in 10% SDS-PAGE gel with conventional composition. Corneal lysates were separated by electrophoresis at 200 V for 100 min. As a positive control, 10 μg of MMP-9 recombinant protein (ab168863, Abcam) was loaded. After gel electrophoresis, the gels were washed with zymogram renaturing buffer (Invitrogen) at room temperature with shaking for 1 h twice. Following the renaturing step, gels were immersed in zymogram developing buffer (Invitrogen) for 30 min at room temperature with shaking. Then gels were incubated in fresh zymogram developing buffer for 48 h at 37 °C with shaking. Fully developed gels stained with colloidal blue staining kit (Invitrogen). The images of stained gels captured with a Bio 5000 scanner (Microtek). The quantification of MMP-9 activity was analyzed with ImageJ open source software [53].

### 4.13. Quantitative Real-Time RT-PCR (qRT-PCR)

Total RNA of CAI cornea was extracted with TRIzol (Invitrogen). In brief, each cornea was immersed in 40 μL of ice-chilled TRIzol and ground with a disposable homogenizer (Biomasher II, 890863, LMS Co.; Tokyo, Japan). The concentration of RNA was measured with Nanodrop^TM^ 2000 (Thermo Fisher Scientific), and 1 μg of RNA templates were used for cDNA synthesis. Generation of cDNA was performed with the reverse transcription reaction kit (iScript^®^ cDNA synthesis kit, Bio-Rad; Hercules, CA, USA). For qRT-PCR, SYBR Green mixture (iQ^TM^ SYBR^®^ Green Supermix, Bio-Rad) and iCycler PCR thermocycler (Bio-Rad) with target gene mRNA template-specific primer sets which designed from GenScript (Piscataway, NJ, USA). The sequence of primers are as follows: α-SMA (5′-GCTATTCAGGCTGTGCTGTC-3′ and 5′-GTTGTGAGTCACGCCATCTC-3′), GAPDH (5′-AAGGCTGTGGGCAAGGTCAT-3′ and 5′-TTTCTCCAGGCGGCATGTCA-3′), and TGF-β1 (5′-GGCTACCATGCCAACTTCTG-3′ and 5′-CGTAGTAGACGATGGGCAGT-3′). The levels of mRNA were normalized with the mRNA level of glyceraldehyde 3-phosphate dehydrogenase (GAPDH), as previously reported [54].

### 4.14. Statistical Analysis

All data are displayed as means ± standard deviation (SD). The normality of data distribution was determined with Shapiro–Wilk test. For parametric data, a one-way analysis of variance (ANOVA) was performed with following post-hoc Tukey’s test used to compare multiple groups. For non-parametric data, Kruskal-Wallis test was performed with the following post-hoc Conover test used to compare multiple groups. MedCalc software version 18.11.6 (MedCalc; Mariakerke, Belgium) was used for all statistical analysis. A *p*-value < 0.05 was considered to be statistically significant.

## Supplementary Materials

Supplementary materials can be found at https://www.mdpi.com/1422-0067/21/8/2990/s1. Supplementary Figure S1: SP-8356 inhibits alkali burn-induced corneal haze at 2-week after alkali burn. (**A**) Schematic diagram for taking representative images of opaque region of flat-mounted cornea. (**B**) Representative images of corneal haze were taken at 2 weeks after alkali burn. The vertically stacked images were taken from the center of cornea (ROI 5). Scale bar, 100 μm (magnification, 100×). (**C**) Summation of corneal opacity grade among five ROIs of four CAI groups at 2 weeks after alkali burn (*n* = 6 for saline, *n* = 9 for HA, *n* = 14 for SP-8356/HA, *n* = 9 for PA). All values are shown as means ± SD (* *p* < 0.05 vs. saline. ** *p* < 0.01 vs. saline. ^#^ *p* < 0.05 vs. HA. ^##^ *p* < 0.01 vs. HA); Supplementary Figure S2: SP-8356 suppresses the myofibroblast population and MMP activity at 2-week after alkali burn. (**A**) Effect of SP-8356 on the relative mRNA level of αSMA (PBS; 0.2× phosphate-buffered saline, SP-8356; 0.933 mM SP-8356 dissolved in 0.2× phosphate-buffered saline, *n* = 10 for sham, *n* = 6 for PBS, *n* = 8 for SP-8356, *n* = 9 for PA). The value of mRNA levels is shown as means ± SD (* *p* < 0.05 vs. PBS. § *p* < 0.05 vs. PA). (**B**) Representative images of MMP9 gelatin acrylamide gel zymography. (C) Quantitative analysis of the relative level of MMP9 activity in whole corneal lysates (*n* = 10 for sham, *n* = 7 for PBS, *n* = 9 for SP-8356, *n* = 7 for PA). MMP9 activity values are shown as means ± SD (* *p* < 0.05 vs. PBS. *** *p* < 0.05 vs. PBS).
