# Supplementary material for: A Novel CD147 Inhibitor, SP-8356, Attenuates Pathological Fibrosis in Alkali-Burned Rat Cornea
Source: Int J Mol Sci. 2020 Apr 23;21(8):2990. doi: 10.3390/ijms21082990 (PMC7215672; doi:10.3390/ijms21082990)
Supplement: Supplementary file 1 [file ijms-21-02990-s001.pdf]

Supplementary Material

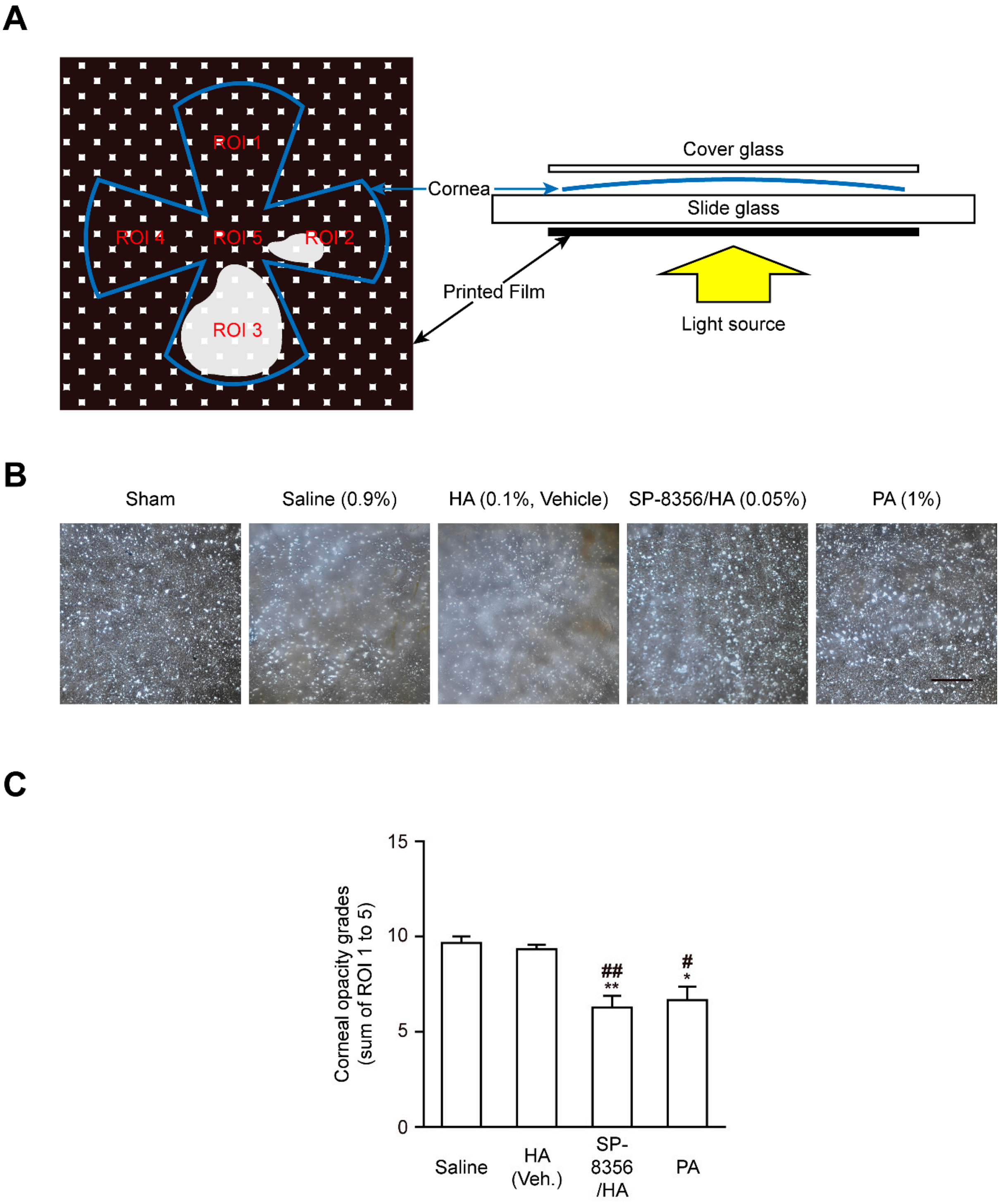

Supplementary Figure S1. SP-8356 inhibits alkali burn-induced corneal haze at 2-week after alkali burn. (**A**) Schematic diagram for taking representative images of opaque region of flat mounted cornea. (**B**) Representative images of corneal haze were taken at 2 weeks after alkali-burn. The vertically stacked images were taken from the center of cornea (ROI 5). Scale bar, 100 μm (magnification, 100×). (**C**) Summation of corneal opacity grade among five ROIs of four CAI groups at 2 weeks after alkali burn (n = 6 for saline, n = 9 for HA, n = 14 for SP-8356/HA, n = 9 for PA). All values are shown as means ± SD (* $p < 0.05$ *vs.* saline. ** $p < 0.01$ *vs.* saline. $^{\#}$ $p < 0.05$ *vs.* HA. $^{\#\#}$ $p < 0.01$ *vs.* HA).

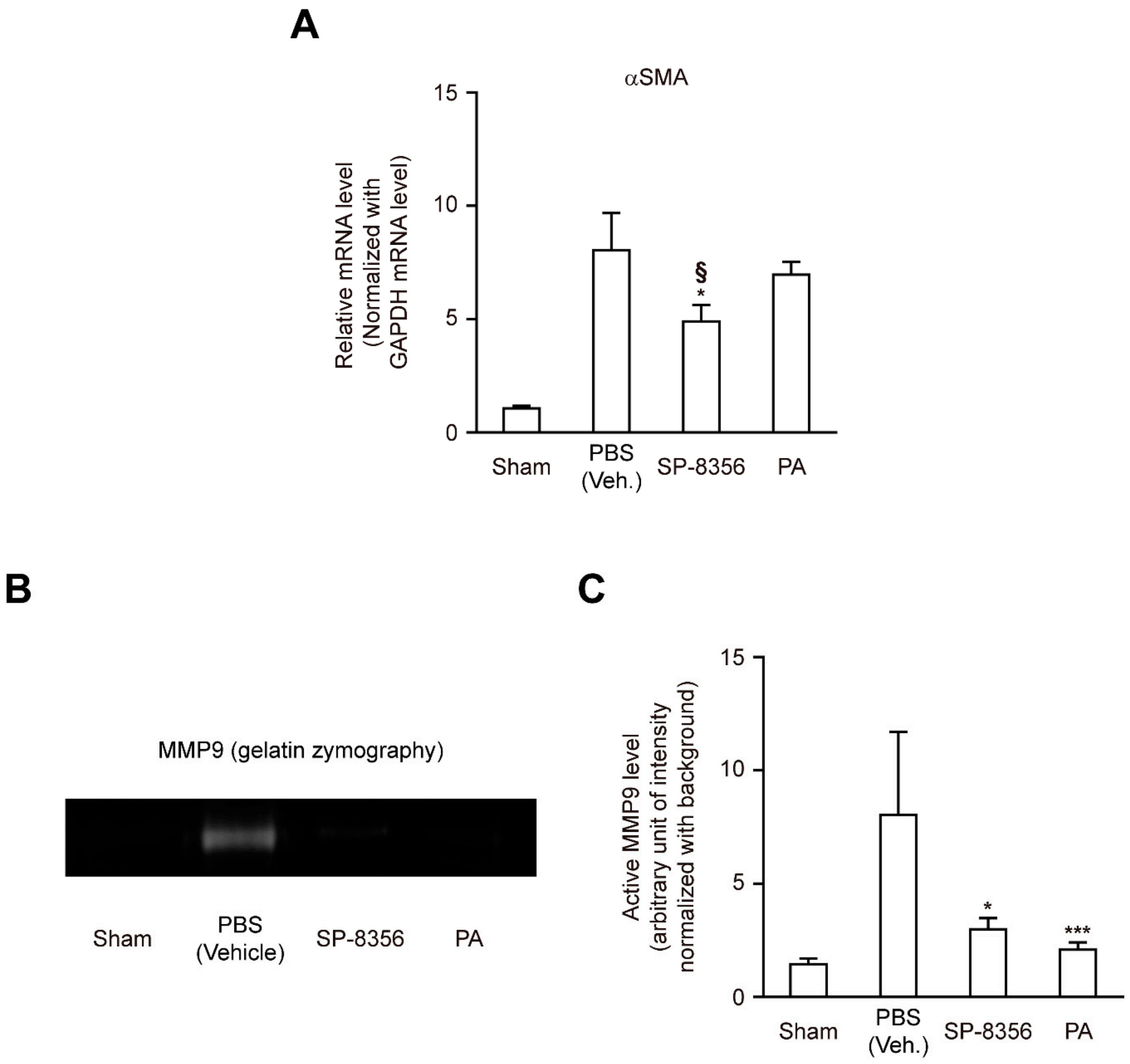

Supplementary Figure S2. SP-8356 suppresses the myofibroblast population and MMP activity at 2-week after alkali burn. (**A**) Effect of SP-8356 on the relative mRNA level of αSMA (PBS; 0.2× phosphate-buffered saline, SP-8356; 0.933 mM SP-8356 dissolved in 0.2× phosphate-buffered saline, n = 10 for sham, n = 6 for PBS, n = 8 for SP-8356, n = 9 for PA). The value of mRNA levels is shown as means ± SD (*$p < 0.05$ *vs.* PBS. §$p < 0.05$ *vs.* PA). (**B**) Representative images of MMP9 gelatin acrylamide gel zymography. (C) Quantitative analysis of the relative level of MMP9 activity in whole corneal lysates (n = 10 for sham, n = 7 for PBS, n = 9 for SP-8356, n = 7 for PA). MMP9 activity values are shown as means ± SD (* $p < 0.05$ *vs.* PBS. *** $p < 0.05$ *vs.* PBS).
